# The use of phosphorescence oxygen analyzer to measure the effects of rotenone and 1-methyl-4-phenylpyridinium on striatal cellular respiration in C57BL6 mice

**DOI:** 10.1016/j.heliyon.2021.e07219

**Published:** 2021-06-05

**Authors:** Mariam Al Shamsi, M.Emdadul Haque, Allen Shahin, Sami Shaban, Abdul-Kader Souid

**Affiliations:** a Department of Microbiology and Immunology, UAE University, College of Medicine and Health Sciences, Alain, P.O. Box 17666, Abu Dhabi, United Arab Emirates; b Department of Biochemistry and Molecular Biology, UAE University, College of Medicine and Health Sciences, Alain, P.O. Box 17666, Abu Dhabi, United Arab Emirates; c Department of Medical Education, UAE University, College of Medicine and Health Sciences, Alain, P.O. Box 17666, Abu Dhabi, United Arab Emirates; d Department of Pediatrics, UAE University, College of Medicine and Health Sciences, Alain, P.O. Box 17666, Abu Dhabi, United Arab Emirates

**Keywords:** Neurons, Basal ganglia, Substantia nigra, Parkinson's disease, Cellular bioenergetics, Cellular respiration

## Abstract

**Background:**

We have previously reported on the use of the phosphorescence oxygen analyzer for measuring spinal cord cellular respiration. This analytical tool is used here to investigate the effects of two inhibitors of NADH:ubiquinone oxidoreductase, rotenone and 1-methyl-4-phenylpyridinium, on cellular respiration in striatal tissue. Both neurotoxins can induce Parkinson's disease-like symptoms, and have been used to study this disease in animals. Our hypothesis is that striatal cellular respiration is a sensitive biomarker for the adverse effects of toxins, and the phosphorescence oxygen analyzer can be used as a screening tool for this purpose.

**Methods:**

Striatal fragments were collected from C57BL6 mice and immersed in Pd phosphor solution [phosphate-buffered saline, 3.0 μM ‘Pd(II)-*meso*-tetra (sulfophenyl) tetrabenzoporphyrin’ and 0.5% fat-free albumin, with and without 5.0 mM glucose]. The sample was transferred to a glass vial containing 2-mL Pd phosphor solution. The vial was sealed from air and placed in the instrument that measures dissolved oxygen as function of time. Immunoblots of the studied tissue were positive for the dopamine neuronal cell biomarker tyrosine hydroxylase.

**Results:**

Striatal oxygen consumption was linear with time, exhibiting zero-order kinetics of oxygen reduction by cytochrome oxidase. Cyanide sensitive respiration was ≥90%, confirming oxygen was reduced by cytochrome oxidase. The rate of respiration increased by ~2-fold in the presence of glucose. Striatal oxygen consumption in the presence of rotenone or 1-methyl-4-phenylpyridinium was exponential, demonstrating impaired respiration.

**Conclusion:**

Striatal cellular mitochondrial oxygen consumption was impaired by the studied inhibitors of complex I of the respiratory chain. This effect is expected to deplete NAD+ (oxidized nicotinamide adenine dinucleotide), a principle driver of glycolysis. *In vivo* studies are required to determine if these toxin-induced metabolic derangements contribute to the development of sporadic Parkinson's disease. This analytic tool can be used to screen environmental toxins for their *in vitro* effects on the striatum.

## 1 Introduction

Parkinson's disease is a progressive neurodegenerative disorder of the central nervous system, which mainly affects our voluntary movements. Its motor symptoms of anomalous posture, tone and movement are a consequence of degeneration of dopaminergic neurons in the substantia nigra of the midbrain that leads to reduced amounts of dopamine neurotransmitters in the striatum [1, 2]. The cause of the disease is mostly unknown; however, available data suggest that exposures to environmental toxicants, such as heavy metals (e.g., manganese, lead, and mercury), illicit drugs (e.g., amphetamine and cocaine), and pesticides (e.g., rotenone [organophosphate], dieldrin [an organochloride], and 1-methyl-4-phenyl1,2,3,6-tetrahydropyridine [MPTP, CAS 28289-54-5]) may produce Parkinson's disease like symptoms [1]. Currently, there are no analytic tools that screen for the adverse outcomes (e.g., mitochondrial dysfunction) of environmental substances on the striatum (i.e., identifying a ‘*potential initiating event*’ or ‘*key event relationship*’) [3]. This preliminary study investigates the use of the phosphorescence oxygen analyzer that measures ‘striatal cellular respiration’ status for this purpose.

Our previous study on experimental autoimmune encephalomyelitis (EAE) has shown that the phosphorescence oxygen analyzer can be used to measure spinal cord cellular respiration in rodents [4]. The same instrument is used here to study the effects of rotenone (CAS 83-79-4) and MPP+ (1-methyl-4-phenylpyridinium: CAS 39794-99-5) on striatal cellular respiration in mice. Both compounds can cause Parkinson's disease-like symptoms, and have been widely used to study the disease in animal models [5, 6].

Rotenone, a pesticide used for crop cultivation, is a well-known reversible/competitive inhibitor of NADH:ubiquinone oxidoreductase. It blocks the transfer of electrons from the iron-sulfur clusters of the enzyme to ubiquinone [5]. MPTP, on the other hand, is a fat-soluble toxin that enters into glial cells and metabolizes to the water-soluble/active metabolite MPP+. The latter uses dopamine transporters of the dopamine-producing neurons for its intracellular uptake [6]. MPP+ is also known to inhibit NADH:ubiquinone oxidoreductase, similar to rotenone [6].

Our hypothesis here is that cellular respiration is a useful biomarker for studying the adverse effects of neurotoxins on the striatum. The main aim of this study is to use the phosphorescence oxygen analyzer to measure striatal cellular respiration in the presence and absence of neurotoxins that are known to cause symptoms similar to Parkinson's disease [7, 8, 9].

## 2 Materials and methods

### 2.1 Chemicals and reagents

Pd(II) meso-tetra (sulfophenyl) tetrabenzoporphyrin sodium salt (C_60_H_32_N_4_Na_4_O_12_PdS_4_; catalog #T41161) was purchased from Frontier Scientific (Logan, UT, USA). Its solution (2.5 mg/mL, or 2.0 mM) was dissolved in dH_2_O and stored at -20 °C. All other reagents were purchased from Sigma-Aldrich (St. Louis, MO). Glucose oxidase (10 mg/mL) was dissolved in H_2_O and stored at -20 °C. Rotenone (2.0 mM) was dissolved in absolute ethanol and stored at -20 °C; its final concentration was determined using molar extinction coefficient at 294 nm of 19,200. 1-Methyl-4-phenylpyridinium iodide (MPP+, molecular weight 297.13) was purchased from Sigma Aldrich, USA. The stock solution of MPP+ (50 mM) was prepared by dissolving 29.71 mg in 2.0 mL sterile dH_2_O; aliquots of the solution were stored at -80 °C.

### 2.2 Animals

C57BL6 male mice (13 weeks old, about 20 g weight) were housed at 22 °C with 60% humidity and 12-h light-dark cycles. Rodent chow and filtered water were provided *ad libitum*. The study was approved from the UAE University Animal Research Ethics Committee (Ref. ERA-2019-6026). Diethyl ether was used for anesthesia.

### 2.3 Striatal tissue collection

The studied mice were not treated in any way prior to striatal tissue collection. The skin and skull were cut to expose the whole brain, which was then rapidly removed and placed in a freshly prepared Pd phosphor solution: Phosphate-buffered saline (PBS: 137 mM NaCl, 2.7 mM KCl, 4.3 mM Na_2_HPO_4_, and 1.4 mM KH_2_PO_4_, *p*H 7.4), 3.0 μM Pd phosphor, 0.5% fat-free albumin, with and without 5.0 mM glucose. The brain region that contained the striatum was dissected. The specimen weight was determined prior to placing it in the oxygen measuring vial that contained 2.0 mL of the Pd phosphor solution. A small portion of the specimen was stored at -80 °C for Western blot analysis.

### 2.4 Western blot analysis of the studied tissue

The tissue specimens were analyzed as previously described [10]. Briefly, the samples were homogenized in a glass homogenizer using 5.0 μL per mg of Radio-immunoprecipitation Assay Buffer (20–188 Millipore) that contained phosphatase and protease inhibitors (Thermo Scientific, Rockford, IL, USA). The homogenates were centrifuged at 4 °C (14,000 rpm for 10 min). Total protein content in the whole cell lysates was determined using the bicinchoninic acid protein assay (Thermo Scientific, Rockford, IL, USA). Twenty μg of the whole cell lysate was separated by electrophoresis, which was then transferred to PVDF (polyvinylidene difluoride) membrane. After blocking in 5% skimmed milk for one hour at 25 °C, the blot was incubated overnight at 4 °C with the mouse monoclonal anti-tyrosine hydroxylase (TH) antibody with a dilution of 1:1000 (ImmunoStar, WI, USA). After washing with PBS-T, the membrane was probed for one hour at 25 °C with horseradish peroxidase (HRP)-conjugated goat anti-mouse secondary antibody with a dilution of 1:20,000 (Jackson ImmunoResearch, PA, USA). The bands of interest were visualized using a West Pico Chemiluminescence kit (Thermo Scientific, Rockford, IL, USA). The blots were stripped and re-probed for GAPDH (1:1000; polyclonal Rabbit, Cell Signaling Technologies, USA) as a loading control [10]. Only samples positive for TH were used in this study.

### 2.5 Oxygen measurement

Our previously described phosphorescence oxygen analyzer was used to monitor oxygen consumption at 25 °C in the studied striatal fragments [4]. The analysis utilized a previously described software programs [11]. The phosphorescence decay rate was calculated using the previously described equations [12]. In reaction vials sealed from air, oxygen concentration decreased linearly with time (zero-order kinetics); the rate (*k*, in μM O_2_ min^−1^) was thus the negative of d[O_2_]/d*t*. The calibration reactions utilized glucose oxidase (D-glucose + O_2_ → D-glucono-δ-lactone + H_2_O_2_) and β-glucose. Dissolved oxygen in the solution was also depleted by the addition of glucose oxidase plus β-glucose [4]. Potassium cyanide (KCN, added from a stock prepared in dH_2_O immediately before use) inhibited respiration, confirming oxygen was consumed in cytochrome oxidase.

## 3 Results

### 3.1 Striatal cellular respiration

**Figure 1:**
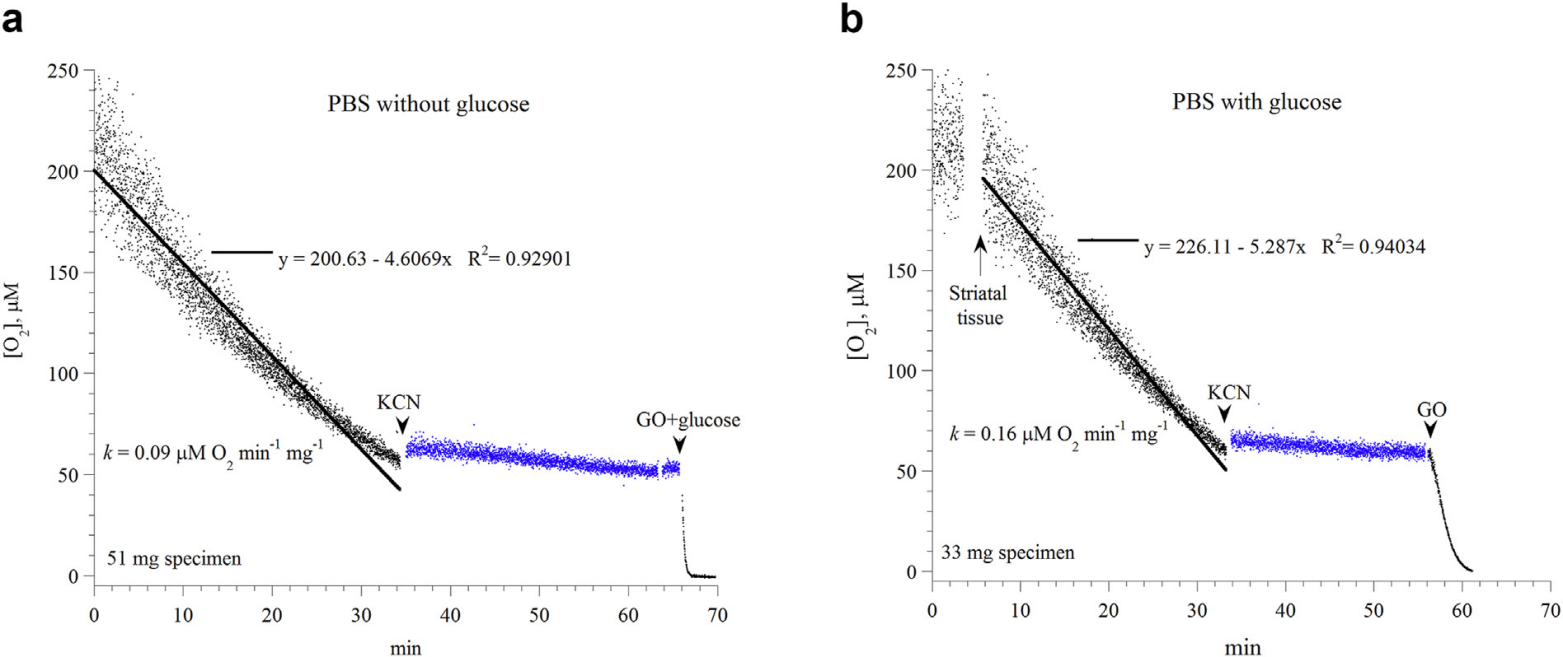
*Striatal cellular respiration*. Representatives of three independent experiments are shown. *Panel a*: A striatal specimen (51 mg) was placed in the Pd phosphor solution (PBS, as described in Methods) without glucose (i.e., the oxidations were fueled by endogenous nutrients only). *Panel b*: A striatal specimen (33 mg) was placed in the Pd phosphor solution plus 5.0 mM glucose (i.e., the oxidations were fueled by endogenous nutrients plus added glucose). The lines are linear fits. The additions of potassium cyanide (KCN) and glucose oxidase (GO) are shown. KCN halted ≥90% of the decline of oxygen concentration with time, confirming oxygen was consumed by cytochrome oxidase. Glucose oxidase (GO) catalyzed the reaction: D-glucose + O_2_ → D-glucono-δ-lactone + H_2_O_2_. The values of *k* (in μM O_2_ min^−1^ mg^−1^) were set as the negative of the slopes of the linear equations (as shown). Noise level over three hours was negligible (not shown). The mice were sacrificed at minute zero.

Striatal cellular respiration was studied in the Pd phosphor solution (the PBS solution described above) with and without 5.0 mM glucose. The results are shown in Figure 1a-b. In both conditions, the decline in oxygen concentration was linear with time. The linear fit, however, was better in the presence of glucose than without glucose (*R*^*2*^, 0.94304 versus 0.92901). The rate of respiration (*k*, in μM O_2_ min^−1^ mg^−1^) without glucose (oxidations driven by the endogenous nutrients) slowed down after 28 min, likely reflecting limited nutrients. It was also faster in the presence of glucose than without glucose (*k*, 0.16 versus 0.09). Thus, the presence of glucose (i.e., oxidations driven by both endogenous nutrients and cellular influx of glucose) improved both the rate and linearity of striatal respiration. The overall value of *k* (mean ± SD) in the presence of glucose was: 0.141 ± 0.041 μM O_2_ min^−1^ mg^−1^ (n = 7). The addition of potassium cyanide (KCN) resulted in ≥90% inhibition of respiration. Thus, the ‘cyanide insensitive respiration’ was <10%. These results confirmed that oxygen was reduced by cytochrome oxidase. The addition of glucose oxidase (GO) depleted remaining oxygen in the solution.

It is also well to know that the linear decline of oxygen concentration with time signified zero-order kinetics of oxygen reduction by cytochrome oxidase (i.e., a constant amount of oxygen was consumed per min) and assured a reasonable striatal tissue viability during the measurements.

### 3.2 Effects of rotenone on striatal cellular respiration

**Figure 2:**
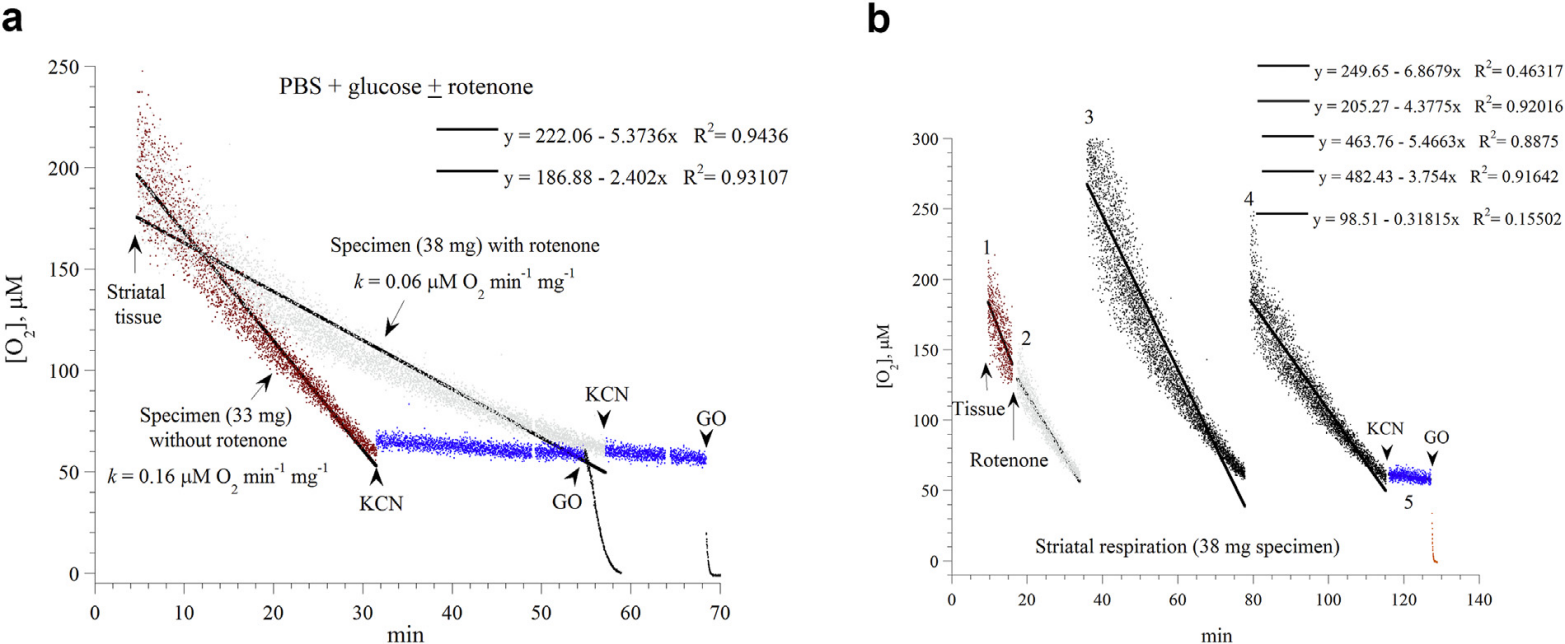
*Effects of rotenone on striatal cellular respiration*. *Panel a*: Representatives of three independent experiments are shown. Striatal specimens were placed in the Pd phosphor solution plus 5.0 mM glucose with and without 10 μM rotenone. The lines are linear fits. The additions of potassium cyanide (KCN) and glucose oxidase (GO) are shown. The values of *k*, in μM O_2_ min^−1^ mg^−1^, were set as the negative of the slopes of the shown linear equations. The mice were sacrificed at minute zero. *Panel b*: Repetitive runs of a striatal specimen (38 mg) in the Pd phosphor solution plus 5.0 mM glucose. The addition of 10 μM rotenone to the baseline run (labeled ‘1’) is shown. At an oxygen concentration of about 50 μM, the specimen was transferred to a new vial containing freshly-prepared (air-saturated) Pd phosphor solution without rotenone. This process was then repeated as shown. The additions of KCN and GO to the last run are also shown. The lines are linear fits. The order of the five linear equations corresponds to the labels of the curves (‘1’ to ‘4’).

Striatal respiration was then studied in the Pd phosphor solution supplemented with 5.0 mM glucose with and without the addition of 10 μM rotenone (Figure 2a,b). The rate of respiration (*k*, in μM O_2_ min^−1^ mg^−1^) in the presence of rotenone was slower (0.092 ± 0.022, n = 5, *P* = 0.073), and the curve fitted was more exponential than linear (*R*^*2*^ = 0.96172 versus *R*^*2*^ = 0.93107). Figure 2b shows that the inhibitory effects of rotenone remained relatively stable over about 100 min.

### 3.3 Effects of MPP+ on striatal cellular respiration

**Figure 3:**
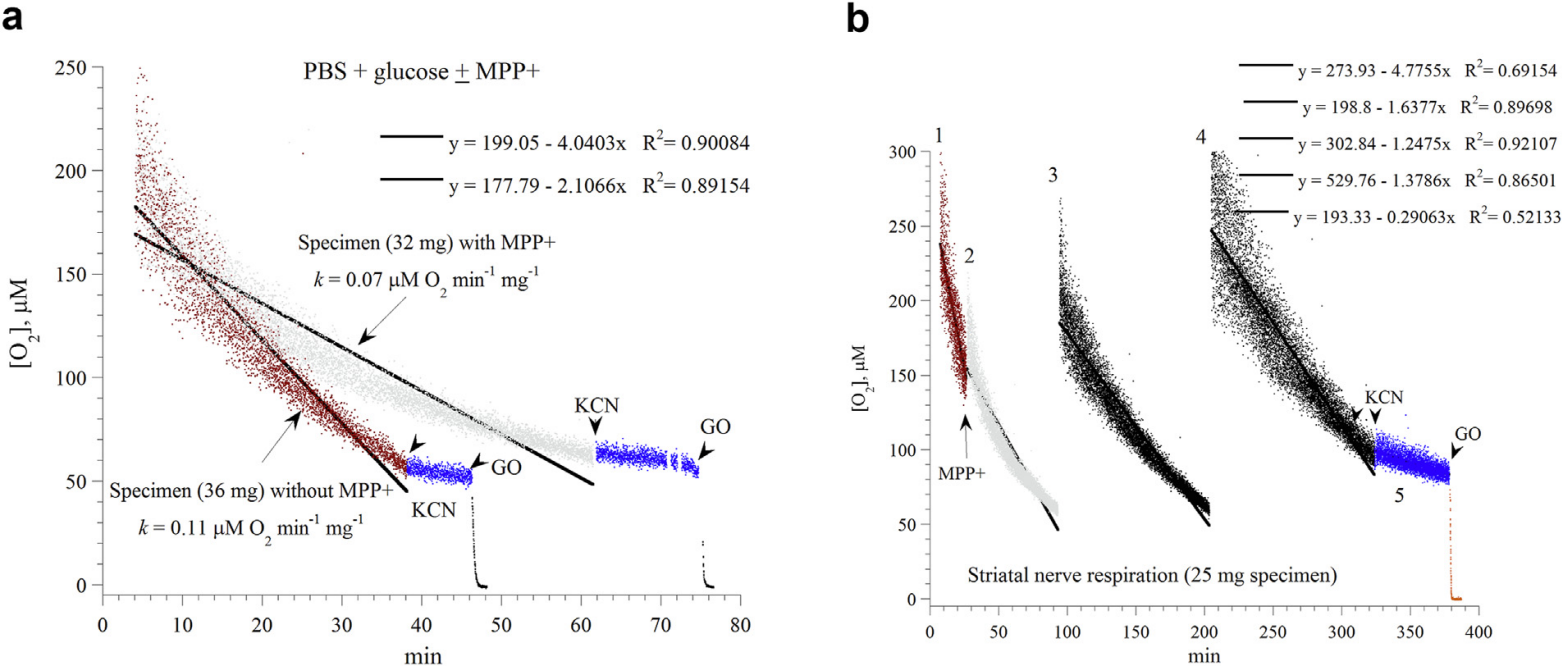
*Effects of MPP+ on striatal cellular respiration*. *Panel a*: Representatives of three independent experiments are shown. Striatal specimens were placed in the Pd phosphor solution plus 5.0 mM glucose with and without 1.25 mM MPP+. The lines are linear fits. The additions of potassium cyanide (KCN) and glucose oxidase are shown. The values of *k*, in μM O_2_ min^−1^ mg^−1^, were set as the negative of the slopes of the shown linear equations. The mice were sacrificed at minute zero. *Panel b*: Repetitive runs of a striatal specimen (25 mg) in the Pd phosphor solution plus 5.0 mM glucose. The addition of 1.25 mM MPP+ to the baseline run (‘1’) is shown. At an oxygen concentration of about 50 μM, the specimen was transferred to a new vial containing freshly-prepared (air-saturated) the Pd phosphor solution without MPP+. This process was then repeated as shown. The additions of KCN and GO to the last run are also shown. The lines are linear fits. The order of the five linear equations corresponds to the labels of the curves.

Striatal respiration was also studied in the Pd phosphor solution supplemented with 5.0 mM glucose with and without the addition of 1.25 mM MPP+ (Figure 3a,b). The rate of respiration (*k*, in μM O_2_ min^−1^ mg^−1^) in the presence of MPP+ was slower (0.077 ± 0.006, n = 3, *p* = 0.048) and the curve fitting was more exponential rather than linear (*R*^*2*^ = 0.90084 versus *R*^*2*^ = 0.89154). Thus, MPP+ also inhibited the striatal respiration. Figure 3b shows the inhibitory effects of MPP+ remained relatively stable over about 300 min.

### 3.4 Western blot of the studied striatal tissue

**Figure 4:**
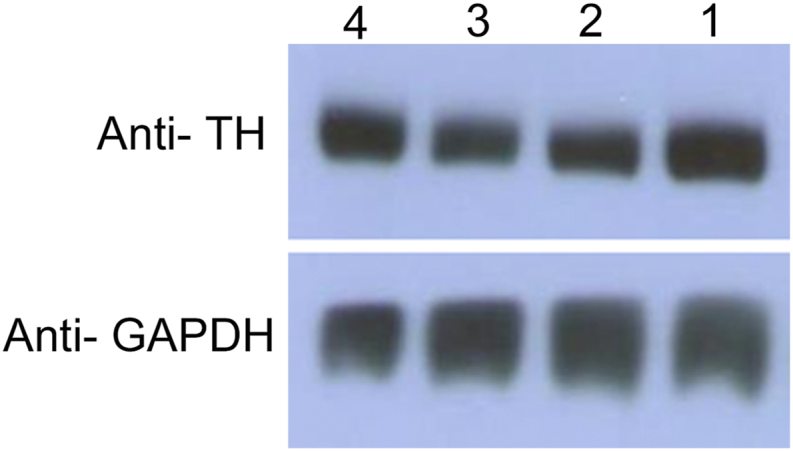
*Western blot of the studied striatal specimen.* The striatal samples were processed as described in Methods (Section 2.4). The blot was probed for the dopamine neuronal cell biomarker tyrosine hydroxylase (TH) and for the loading control glyceraldehyde 3-phosphate dehydrogenase (GAPDH). This study involved only the specimens that were positive for TH (Supplementary Material, Western blot of the striatum-November 05, 2020).

The studied striatal specimens were probed for the dopamine neuronal cell biomarker tyrosine hydroxylase (TH). This study involved only the specimens that were positive for TH (Figure 4).

## 4 Discussion

The potential link between Parkinson's disease and mitochondrial poisons, such as rotenone and MPTP has been extensively studied [5, 6]. These mitochondrial toxins can alter the cellular bioenergetics of the midbrain, causing substantial changes in the dopaminergic neurons [7]. In this study, we used these two respiratory inhibitors to measure their direct effects on striatal oxygen consumption. The previously described phosphorescence oxygen analyzer was used for this purpose [4].

Striatal cellular respiration was linear with time and was inhibited by cyanide, confirming the expected zero-order kinetics of oxygen reduction by cytochrome oxidase in the studied viable tissue. Striatal cellular respiration is also inhibited by rotenone and MPP+, confirming the adverse effects of these known inhibitors of NADH:ubiquinone oxidoreductase on the striatum *in vitro*. This metabolic event (inhibition of NADH:ubiquinone oxidoreductase) is expected to block NADH (reduced nicotinamide adenine dinucleotide) oxidation, thus, depleting cellular NAD+ (oxidized nicotinamide adenine dinucleotide). Briefly, NADH generated in glucose metabolism, especially in the citric acid cycle must be oxidized to NAD+ by passing its electrons to the electron transport chain and finally to oxygen in the mitochondrial cytochrome oxidase (Complex IV). Inability to regenerate the NAD+ would halt all energy producing reactions, especially in the citric acid cycle [13].

NAD+, an electron carrier in over 100 major catabolic reactions, has been recently recognized as a principle driver of the glycolysis and citric acid cycle, and a fundamental regulator of the energy metabolism (a bioelectric wire for ATP [adenosine triphosphate] synthase) [13]. Likewise, metabolic perturbations affecting NAD+ have been linked to several human diseases, including Parkinson's and Alzheimer's diseases [14].

Each molecule of NAD+ can accept one hydride ion (a proton and two electrons,**:**H^**-**^) in the nicotinamide ring (reversible reduction) [15]. The resulting reducing equivalent NADH then wires these electrons to NADH:ubiquinone oxidoreductase (dehydrogenation/oxidation), generating an electrochemical potential (electromotive force) across the inner mitochondrial membrane that drives ATP synthesis (phosphorylation). Consequently, inhibitors of NADH:ubiquinone oxidoreductase, such as rotenone and MPP+ are expected to deplete both NAD+ and ATP [16, 17].

The ratio of NAD+ to NADH in the cell is typically high, favoring hydride transfers by over 200 known oxidoreductases in the main metabolic pathways (e.g., oxidations of pyruvate, fatty acids, α-keto acids) to form NADH [18]. Thus, cellular NAD+ is central in the electron transport that supports of oxidative phosphorylation [15]. Future studies, however, are needed to determine whether this metabolic derangement (cellular depletion of NAD+) dominates in both sporadic and genetic forms of Parkinson's disease [14]. Interestingly, nicotinic acid (vitamin niacin) deficiency can affect all NAD+ dependent oxidoreductases, causing the serious human disease pellagra (dementia, diarrhea, and dermatitis) [18].

Using the above described method, striatal tissue can be investigated by *in vitro* exposure of viable tissue to toxins, as shown here. The same approach can also be used by collecting striatal tissue following *in vivo* treatment of the animal. Advantages of using this analyzer include its simplicity, low-cost, and ability to monitor respiration over several hours. Its disadvantages, however, include studying one sample at a time and needs for viable (freshly-collected) tissue.

In conclusion, we show here that the phosphorescence oxygen analyzer is useful in evaluating the adverse effects of the studied two metabolic poisons on the striatum. Future studies are needed to explore its use as a screening tool for studying the effects of a wide-range of environmental compounds on the striatum.

## Declarations

### Author contribution statement

Mariam Al Shamsi: Conceived and designed the experiments; Performed the experiments; Analyzed and interpreted the data; Wrote the paper.

M Emdadul Haque: Performed the experiments; Analyzed and interpreted the data; Wrote the paper.

Allen Shahin, Sami Shaban: Contributed reagents, materials, analysis tools or data.

Abdul-Kader Souid: Conceived and designed the experiments; Analyzed and interpreted the data; Wrote the paper.

### Funding statement

This work was supported by the College of Medicine & Health Sciences, UAE University (A grant).

### Data availability statement

Data included in article/supplementary material/referenced in article.

### Declaration of interests statement

The authors declare no conflict of interest.

### Additional information

No additional information is available for this paper.

## Appendix A Supplementary data

Western blot of the striatum-November 05_spl_ 2020 Western blot of the striatum-November 05_spl_ 2020
