# Supplementary material for: The use of phosphorescence oxygen analyzer to measure the effects of rotenone and 1-methyl-4-phenylpyridinium on striatal cellular respiration in C57BL6 mice
Source: Heliyon. 2021 Jun 5;7(6):e07219. doi: 10.1016/j.heliyon.2021.e07219 (PMC8203712; doi:10.1016/j.heliyon.2021.e07219)
Supplement: Western blot of the striatum-November 05_spl_ 2020 [file mmc1.pdf]

Western blot of the striatum-Nov 5, 2020

- The tissue processed for WCL (1X RIPA+1X PPI)
- 20ug was loaded
- Membranes were probed against TH Ab and anti GAPDH.

Different exposures

Anti- TH

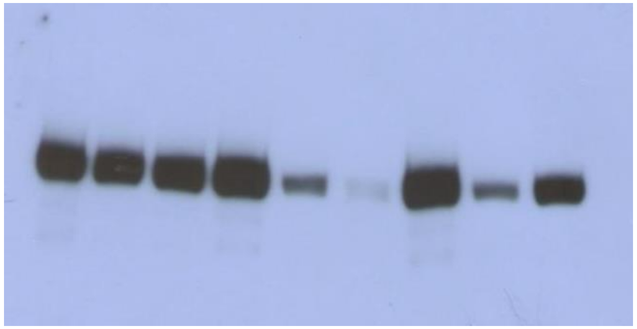

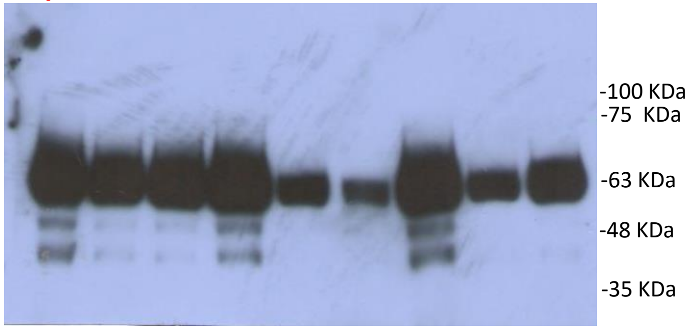

Anti- TH

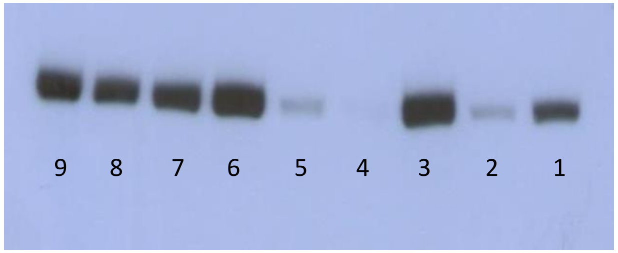

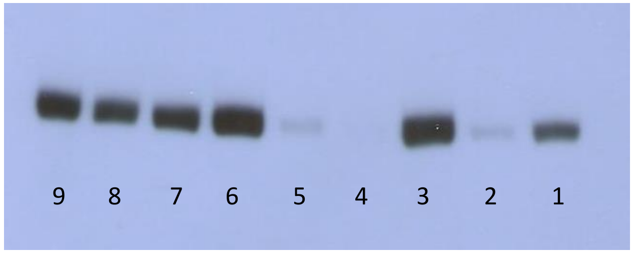

Anti- GAPDH

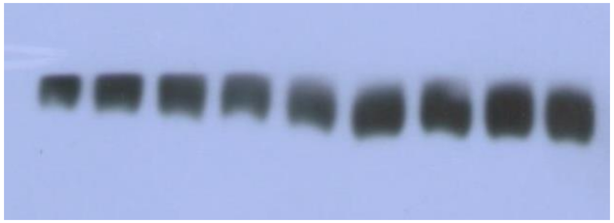

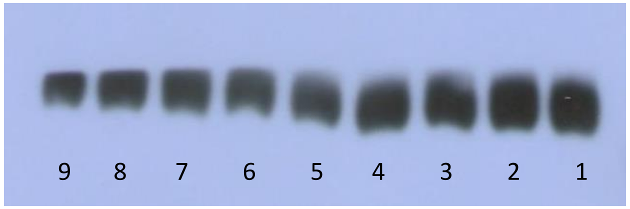

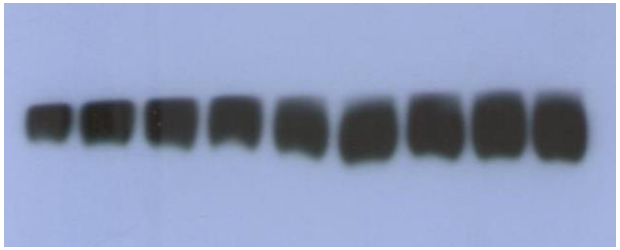

Anti- GAPDH

1. Sample 26/10/20
2. Sample 27/10/20
3. Sample 28/10/20 A
4. Sample 28/10/20 B
5. Sample 1/11/20
6. Sample 2/11/20 A
7. Sample 2/11/20 B
8. Sample 3/11/20 A
9. Sample 3/11/20 B
